# Pαx6 Expression in Postmitotic Neurons Mediates the Growth of Axons in Response to SFRP1

**DOI:** 10.1371/journal.pone.0031590

**Published:** 2012-02-16

**Authors:** Alvaro Sebastián-Serrano, Africa Sandonis, Marcos Cardozo, Fernanda M. Rodríguez-Tornos, Paola Bovolenta, Marta Nieto

**Affiliations:** 1 Centro Nacional de Biotecnología, Consejo Superior de Investigaciones Científicas, Madrid, Spain; 2 Centro de Biologia Molecular Severo Ochoa, Consejo Superior de Investigaciones Científicas-UAM, and CIBER de Enfermedades Raras, Madrid, Spain; University of Dayton, United States of America

## Abstract

During development, the mechanisms that specify neuronal subclasses are coupled to those that determine their axonal response to guidance cues. Pax6 is a homedomain transcription factor required for the specification of a variety of neural precursors. After cell cycle exit, *Pax6* expression is often shut down in the precursor progeny and most postmitotic neurons no longer express detectable levels of the protein. There are however exceptions and high Pax6 protein levels are found, for example, in postmitotic retinal ganglion cells (RGCs), dopaminergic neurons of the olfactory bulb and the limbic system in the telencephalon. The function of Pax6 in these differentiating neurons remains mostly elusive. Here, we demonstrate that Pax6 mediates the response of growing axons to SFRP1, a secreted molecule expressed in several Pax6-positive forebrain territories. Forced expression of *Pax6* in cultured postmitotic cortical neurons, which do not normally express *Pax6*, was sufficient to increment axonal length. Growth was blocked by the addition of anti-SFRP1 antibodies, whereas exogenously added SFRP1 increased axonal growth of Pax6-transfected neurons but not that of control or untransfected cortical neurons. In the reverse scenario, shRNA-mediated knock-down of *Pax6* in mouse retinal explants specifically abolished RGCs axonal growth induced by SFRP1, but had no effect on RGCs differentiation and it did not modify the effect of Shh or Netrin on axon growth. Taken together these results demonstrate that expression of Pax6 is necessary and sufficient to render postmitotic neurons competent to respond to SFRP1. These results reveal a novel and unexpected function of *Pax6* in postmitotic neurons and situate *Pax6* and SFRP1 as pair regulators of axonal connectivity.

## Introduction

The selective response of axons to elongation and guidance cues encountered along their paths enables the precise formation of neuronal circuits and the formation of topographic maps. During development, mechanisms that specify neuronal subclasses are coupled to those that specify their axonal response through the selective expression of transcription factors.

Pax6 is a homeodomain transcription factor expressed in several territories of the developing nervous system, mostly in the proliferative regions containing neural precursors [1]. The vast majority of the neural progeny of these precursors including neurons of the dorsal cortical plate or neural stem cell (NSCs) derived neurons, shut down *Pax6* expression upon exiting the cell cycle. Consequently, very few postmitotic populations express *Pax6*. Notable examples are the retinal ganglion cells (RGCs), the dopaminergic neurons of the olfactory bulb and certain neurons in the basal telencephalon and midbrain [2], [3], [4], [5]. While the role of *Pax6* in neural precursors has been widely explored, revealing a central function in cell fate specification and cell cycle regulation [5], [6], [7], [8], [9], less attention has been paid to its functions in postmitotic neurons, with the major exception of a recent study demonstrating that Pax6 is needed for the survival of dopaminergic neurons in the olfactory bulb [10].

RGCs are one of the best-characterized postmitotic populations that express *Pax6* [11]. *Pax6* expression in both developing and mature RGCs is graded, with higher levels in the ventro-temporal distal cells and lower in the proximal domains [11]. These two differentially expressing *Pax6* populations project to distinct non-overlapping and complementary topographic regions of the superior culliculus and lateral geniculate nucleus (LGN), which suggests that *Pax6* may contribute to control axon targeting. Indeed, RGCs of mice overexpressing *Pax6* show disrupted axonal trajectories and abnormal bundle formation [12], whereas changes in Pax6 expression in the RGCs correlate with axonal regeneration in the optic nerve of lizards and zebrafish [13]. Still, there is no information on whether *Pax6* is directly involved in the control of axon growth and what guidance cues, if any, depend upon its activity.

Secreted Frizzled-Related Protein 1 (SFRP1) is one of the factors known to stimulate the directional growth of RGCs axons in *Xenopus* and chick retina. This activity is independent of Wnt signaling and modulated by extracellular matrix molecules [14], [15]. Mouse *Sfrp1* is expressed in several structures of the embryonic and adult eye and brain, including the pigmented retina, cornea, ciliary bodies, lens epithelium, the prospective thalamus and the proliferative regions of the telencephalon [14], [16], [17], [18], [19]. The SFRP1 distribution often coincides with that of Pax6 or decorates the axonal pathways of Pax6 expressing neurons [14], [16], [17], [18], [19], raising the possibility of a functional relationship between the two molecules.

Here we show that expression of *Pax6* in neuronal postmitotic populations is necessary and sufficient to confer neurons with a positive response to SFRP1. Over-expression of *Pax6* rendered cultured cortical neurons competent to respond to endogenous and exogenous SFRP1. Conversely, knock-down of *Pax6* in mouse retinal explants abolishes RGC axonal growth induced by SFRP1 without affecting RGC differentiation or their axonal response to a different guidance cue like sonic hedgehog (Shh) or Netrin. These results suggest that *Pax6* and SFRP1 are pair regulators of axonal connectivity in the retina and reveal a new function of *Pax6* in the postmitotic populations.

## Results

### 1. *Pax6* induces axonal growth in postmitotic neurons

**Figure 1:**
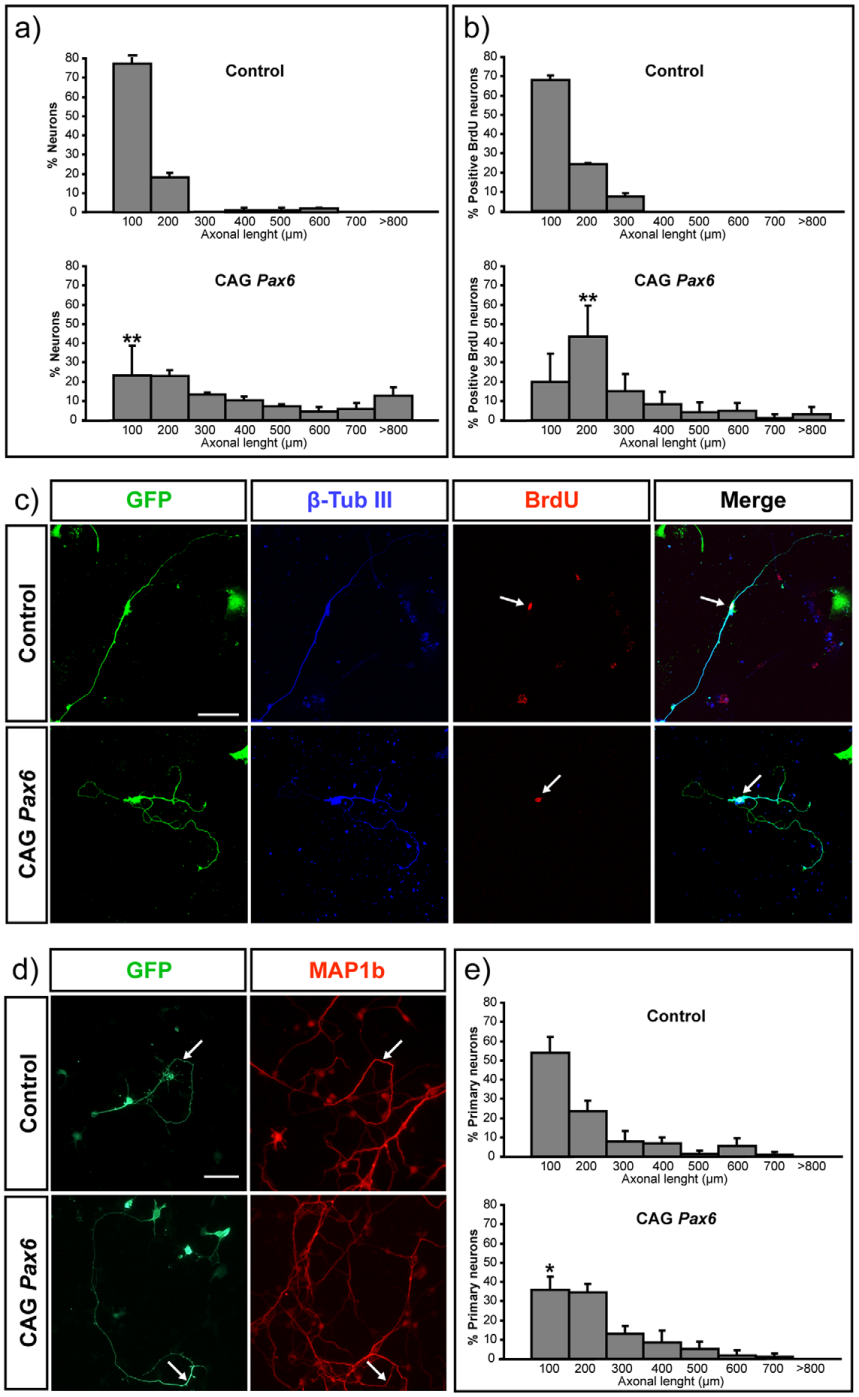
Ectopic expression of *Pax6* stimulates axonal growth in cortical neurons. NSCs were nucleofected with CAG-empty vector or CAG-*Pax6* and co-electroporated with CAG-*GFP*. **a**) The graph shows the percentage of nucleofected neurons with respect to their axonal length after 9 days of differentiation. Over-expression of *Pax6* increments the axonal length compared with control neurons. **b**) The graph shows quantification of the axonal length of a cohort of BrdU positive neurons. **c**) Nucleofected neurons were identified by the specific expression of β-tubulin III and GFP. BrdU staining allowed us to compared neurons of similar birth dates. The effect in axonal growth is unrelated to an early exit from the cell cycle. Arrows point to BrdU stained nuclei. Bar indicates 50 µm. **d**) Cortical primary neurons were also transfected with CAG-empty vector or CAG*-Pax6*, and with CAG-*GFP*. Transfected primary axons were identified by GFP and MAP1b staining. Arrows indicate the distal part of the axon stained by MAP1b. Bar indicates 70 µm. **e**) The graph shows the percentage of primary neurons with respect to their axonal length. Data are expressed as the mean ± SD. (*) p<0.05; (**) p<0.01.

The majority of postmitotic neurons, including those derived from telencephalic *Pax6* positive precursors, such as cerebral cortical neurons or neurons derived from cortical neural stem cells (NSCs), do not express *Pax6* [2] (Fig. S1a). To test the consequences of *Pax6* expression in postmitotic populations, we forced its expression in neurons derived from mouse NSCs. NSCs grown as neurospheres were nucleofected with CAG-empty vector or CAG-*Pax6*, together with a CAG-*GFP* that allowed the visualization of transfected cells. GFP staining demonstrated efficient transfection by FACs cytometry (Fig. S1b) and immunostaining of plated cells confirmed that cells transfected with CAG-*Pax6*, but not those transfected with the empty vector, had nuclear Pax6 protein expression (Fig. S1a). Plated transfected cells were then allowed to differentiate for 9 days to analyze their differentiation and morphology. GFP neurons identified by β-tubulin III expression show an advance differentiated asymmetrical polarized morphology, both in control and Pax6 transfected cells (Fig. 1 c), and the longer neurite was identified as the axon by MAP1b staining (not shown), a selective marker of the distal part of the growing axon [24]. Overexpression of *Pax6* alone was sufficient to promote axonal extension compared to control neurons (Fig. 1a). Quantitative analysis of β-tubulin III positive-projections confirmed a significant increment of the axonal length from neurons transfected with CAG*-Pax6* both at 6 and 9 days of culture (Fig. S2 and Fig. 1a, respectively). Similar results were obtained upon quantification of MAP1b projecting axons (not shown). The total number and extension of the additional neurites was not affected (Figure S1c).

Several reports have shown that *Pax6* over-expression induces precursor cells to exit the cell cycle and differentiate [9], [25]. To discard the possibility that increased axonal length was a consequence of a premature exit from cell cycle and, thus, of an extended time of differentiation, we restricted our study to those cells that were leaving the cell cycle at similar times. To visualize cohorts of nascent neurons, BrdU was added on the second day of culture and extensively washed after 12 h. Analysis of BrdU-positive neurons on the ninth day of culture confirmed that the axons of the *Pax6* expressing population were significantly longer than those of cells transfected with mock vector (Fig. 1b and c). Restricting to this cohort of BrdU neurons correlated with a higher increment in axonal length as expected from the analysis of a more homogenous population (compared Fig. 1a and b). This demonstrates that the effect in axonal growth is unrelated to an early exit from the cell cycle. To strengthen this conclusion, we over-expressed *Pax6* in non-proliferating primary cortical neurons and quantified the length of MAP1b projections, obtaining similar results (Fig. 1d and e), although the increment of outgrowth was less evident than that observed in NCSs, likely owing to the heterogeneity of primary differentiating neurons. Together, these experiments demonstrated that *Pax6* expression in differentiated neurons stimulates process elongation by a cell cycle independent mechanism.

### 2. *Pax6* mediates the axonal response to SFRP1

**Figure 2:**
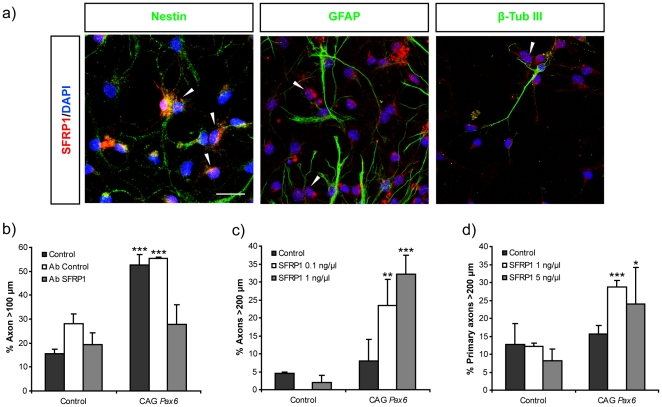
SFRP1 secreted by cortical cells *in vitro* is responsible for the axonal growth observed in *Pax6* over-expressing neurons. **a**) Immunofluorescence shows expression of SFRP1 (red) in differentiating, NSCs cultured for 5 days. Cells were double labeled with anti-nestin, GFAP and β-tubulin III. SFRP1 expression is found in the cytoplasma of Nestin-positive cells or in some with a low GFAP expression (arrowheads). No SFRP1 expression was found in cell with high GFAP levels or in β-tubulin III-positive cells. Bar represents 20 µm. **b**) The graphs show the percent of axons longer than 100 µm upon *Pax6* overexpression in NSCs when cultured in the presence or absence of control or anti-SFRP1 antibodies. Note that antibody against SFRP1 blocks axonal growth stimulated by the ectopic expression of *Pax6*. **c, d**) Response to increasing concentrations of purified recombinant SFRP1 in neurons derived from NSC c) or primary cortical neurons (d). NSCs (c) and primary neurons (d) were transfected with control empty CAG and CAG-*Pax6* vectors and stimulated with different concentrations of SFRP1.Addition of recombinant SFRP1 only stimulates the growth and elongation of axons of neurons ectopically expressing *Pax6*. Data are expressed as the mean ± SD. (*) p<0.05; (**) p<0.01; (***) p<0.001. Number of axons per condition >50 (n = 3).

*SFRP1* is expressed by the neural precursors of the ventricular zone (VZ) of the dorsal telencephalon, which give rise to both cortical neurons and NSCs [2]. We confirmed that neural precursor cells cultivated *in vitro* also express detectable levels of the SFRP1 protein (Fig. 2a). We therefore postulated that SFRP1 produced and secreted in the culture by undifferentiated cells was responsible for the selective axonal growth of *Pax6*-transfected neurons. Addition of antibodies against SFRP1 to the culture media two days after NSCs transfection completely blocked *Pax6*-induced axonal growth, leading to processes with a length undistinguishable from that of control cells (Fig. 2b). This result was specific since addition of control unrelated antibodies had no effect (Fig. 2b). This data indicated that *Pax6* stimulated the growth of NSCs derived neurons in response to endogenous SFRP1 produced in the *in vitro* culture.

To support this conclusion, we next assayed the effects of exogenously added SFRP1 upon forced expression of *Pax6* in primary cortical neurons or neurons derived from NSCs. Purified, recombinant SFRP1 was added to the neuronal culture after transfection and the early axonal response was evaluated after two or three days in cortical neurons and NSCs, respectively. Notably, addition of exogenous SFRP1 to control neurons had no effect on the length of their axons, but stimulated, in a dose dependent manner, the elongation of those belonging to Pax6-positive neurons (Fig. 2c, d and Fig. S3). Therefore, *Pax6* appears to be necessary and sufficient to confer cortical neurons with the competence to respond to SFRP1 of exogenous or endogenous origin.

**Figure 3:**
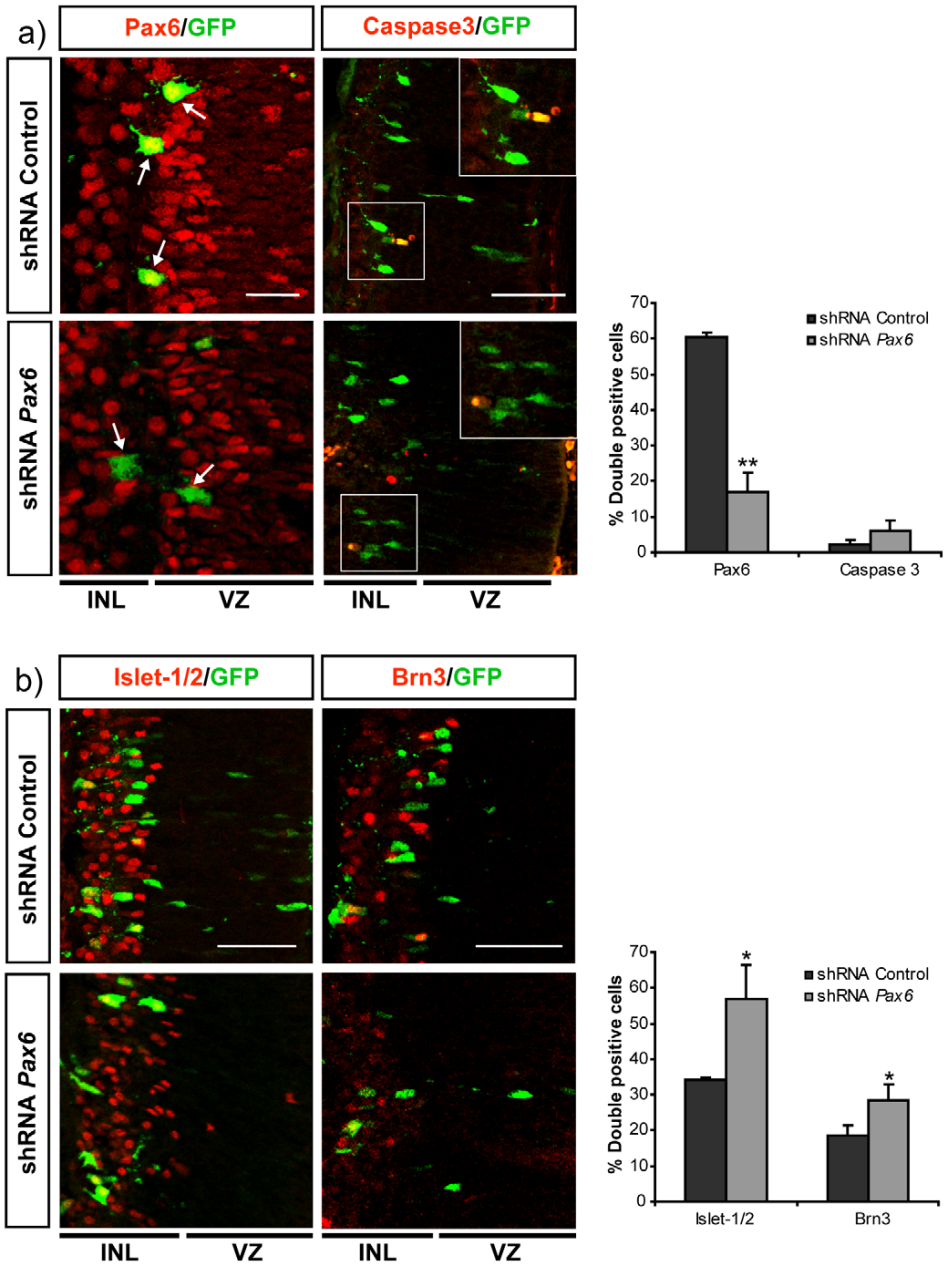
Knock-down of *Pax6* in the embryonic retina does not interfere with the generation of RGCs. Frontal cryostat sections of E19.5 retinas after electroporation at E13.5 with shRNAs constructs control and targeting *Pax6* and immunostained (red) for Pax6 and Caspase 3 (**a**) or Islet-1/2 and Brn3 (**b**) as indicated in the panels. Electroporated cells are visualized with GFP (green). The graphs show the quantification of the proportion of the double positive cells for the indicated marker. Note that shRNA efficiently target Pax6 without inducing cell death (a) or changes in expression of markers for postmitotic RGCs (b). Bar indicates 10 in (a) and 40 µm in (b). Data are expressed as the mean ± SD. (*) p<0.05; (**) p<0.01. Number of axons per condition >60 (n = 3). INL, inner layer; VZ, ventricular zone.

Postmitotic RGCs express Pax6 [4] and their axons respond to SFRP1 [14]. We therefore asked whether this response was similarly dependent on Pax6. To this end we decided to knock-down *Pax6* using shRNA lentiviral constructs. Effective knock-down of the Pax6 protein by the shRNA lentiviral constructs was first evaluated in cultured mammalian CHO cells transfected with *Pax6* (See Methods and Fig. S1d). Selected constructs were thereafter targeted to the retina of E13.5 embryos by *in utero* electroporation. A CAG-GFP plasmid was co-electroporated to visualize targeted cells and their axons. The expression of Pax6 and the fate of the targeted cells were analyzed at E19.5 in histological sections. In retinas targeted with either control or *Pax6* shRNA, the majority of GFP-positive cells were located in the inner layers, corresponding with the normal location of RGCs (Fig. 3a and b). Double staining with anti-Pax6 and -GFP antibodies demonstrated strong expression of Pax6 in GFP-positive control cells but undetectable levels in the majority of cells electroporated with the *Pax6-*shRNA (Fig. 3a). Quantification of the number of GFP and Pax6 positive cells demonstrated that the selected shRNA resulted in efficient down-modulation of *Pax6* in the mouse retina cells (Fig. 3a, graph). No significant changes were observed in the number of apoptotic, Caspase3-positive cells [26] (Fig. 3a). Because *Pax6* is involved in cell fate decisions and cell cycle exit [4], [9], [27] we next analyzed the molecular identity of the GFP electroporated cells using two RGC specific markers: Islet-1/2, that marks most of RGCs [28], and Brn3, which labels only a subpopulation [29]. In control retinas, an important proportion of GFP positive cells showed expression of Islet-1/2 and Brn3, with the expected pattern. Down-regulation of *Pax6* did not block RGC generation but somehow favored the increase of the number of Islet-1/2 positive cells, which was less clearly correlated with that of *Brn3* positive cells (Fig. 3b). This moderated shift in the proportion of RGCs cells might be associated to the reported effects in cell cycle exit [4], [9], [27], rather than on selective cell death, which was invariant (Fig. 3a).

**Figure 4:**
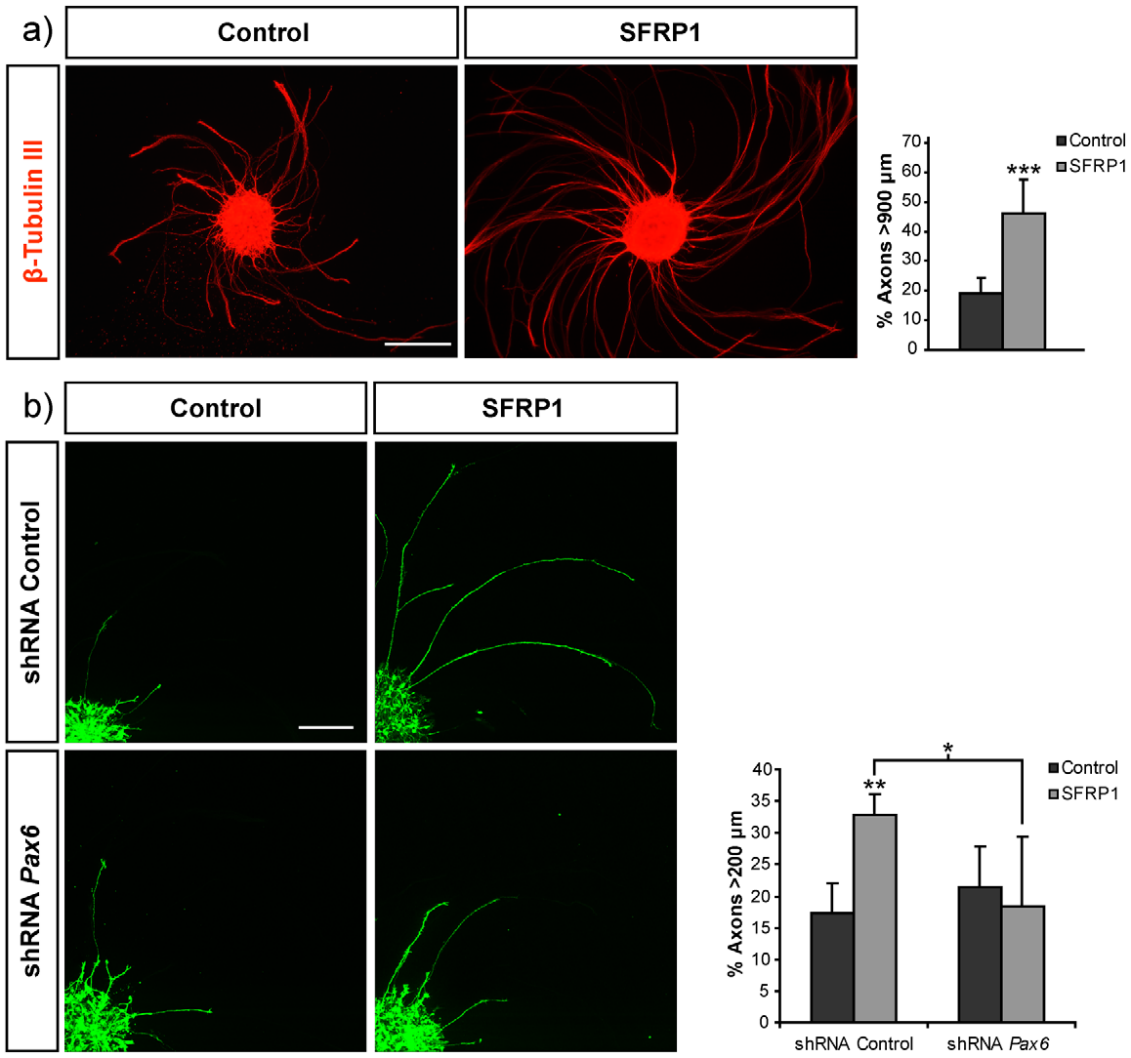
Knock-down of *Pax6* blocks SFRP1 stimulated growth of retinal axons. **a**) Low magnification images (a) and confocal micrographs (b) of explants from control (a) or electroporated retinal explants (b) seeded onto laminin coated coverslip and cultured in the absence or presence of recombinant SFRP1 as indicated in the panels. Explants in (a) were stained with β-tubulin III whereas those in (b) were co-electroporated with CAG-GFP and shRNA control or shRNA targeting *Pax6* and axons were visualized by GFP expression. Graph shows quantification of the proportion of total axon longer than 900 (a) or 200 (b) µm. Note that knocking-down *Pax6* inhibits the axonal response stimulated by SFRP1. Bar indicates 300 (a) and 150 µm (b). Data are expressed as the mean ± SD. (*) p<0.05 comparing stimulated populations; (**) p<0.01 and (***) p<0.001 compared with control.

**Figure 5:**
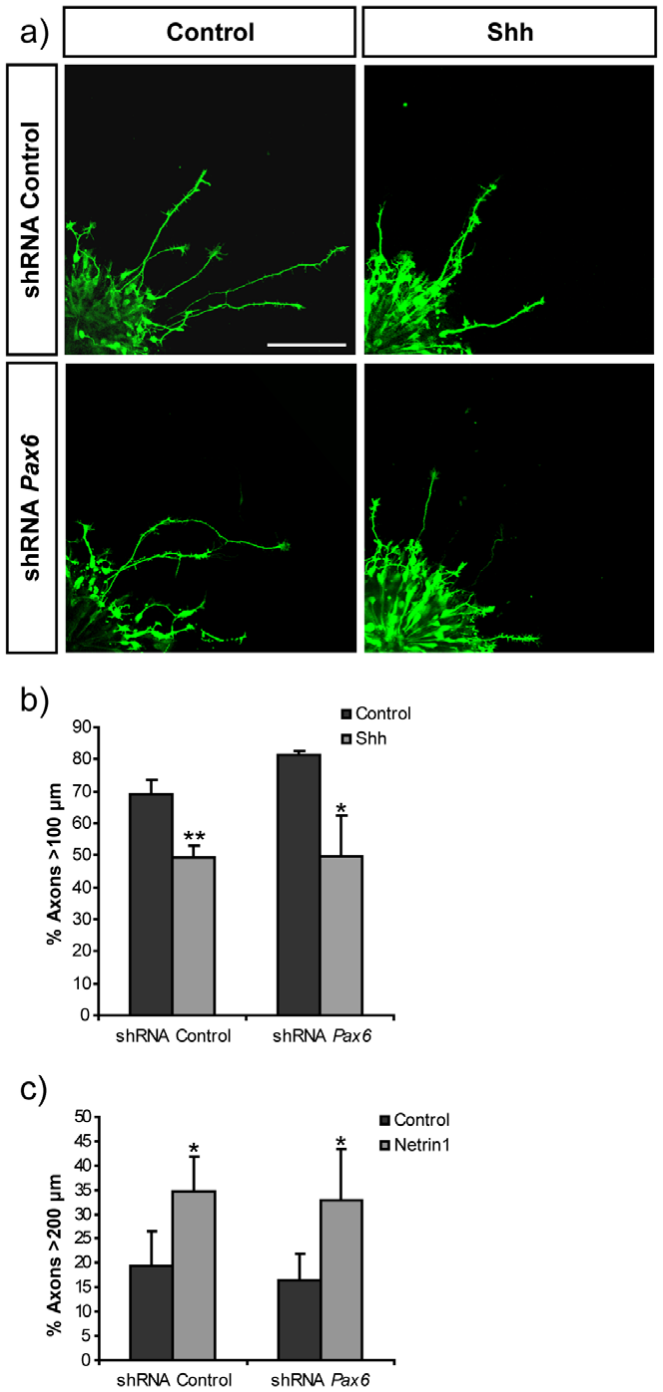
Knock-down of *Pax6* does not affect the response of retinal axons to Shh or Netrin 1. **a**) Micrographs show confocal images of GFP positive axons of retina explants co-electroporated with CAG-GFP and control shRNA or shRNA targeting Pax6. Explants were seeded onto laminin coated coverslip in the presence or absence of recombinant Shh. Bar indicates 100 µm. **b**) Graph shows quantification of the proportion of GFP positive axons longer than 100 µm in the presence or absence of recombinant Shh. **c**) Graph shows quantification of the proportion of GFP positive axons longer than 200 µm in the presence or absence of recombinant Netrin 1. Data are expressed as the mean ± SD. (*) p<0.05; (**) p<0.01.

Given that shRNA-mediated knock-down of *Pax6* in E13.5 retinal precursors did not interfere with RGC generation and organization, we examined the response of *Pax6* deficient RGCs to SFRP1 [14]. Explants from E13.5 retinas electroporated *ex vivo* with control shRNA or shRNA constructs targeting *Pax6* were seeded on laminin-coated coverslips, stimulated with SFRP1 and fixed 24 h after. Staining with anti-β-tubulin III confirmed that, as in chick and *Xenopus* [14], SFRP1 stimulated axonal outgrowth and extension from control mouse retinas, increasing the proportion of axons longer than 900 µm (Fig. 4a). Similarly, addition of SFRP1 to control electroporated retinal explants doubled the proportion of GFP-positive electroporated RGCs with axons longer than 200 µm (Fig. 4b), although the average length was appreciably shorter than that of axons from non-electroporated retinas (compare Fig. 4a and 4b). Likely, this is because targeted cells only account for newly generated RGCs [30]. Knock-down of *Pax6* in RGCs prevented their response to SFRP1 (Fig. 4b) but did not interfere with axonal initiation because the total number of axons per explants was not significantly different in control and *Pax6* shRNA electroporated retinas in the presence or absence of SFRP1 (not shown). Furthermore, in non-stimulated explants, the length of GFP positive axons was similar in both control and *Pax6* shRNA electroporated retinas (Fig. 4b). Notably, axonal growth was restored when knock-down was attempted in the presence of a silent mutant form of *Pax6* resistant to shRNA, excluding off-target effects of the shRNA constructs (not shown). Furthermore, *Pax6* knock-down had no effect on the response of dorsal retinal explants to exogenously added Shh (Fig. 5a and 5b) or Netrin1 (Fig. 5c), additional axon guidance cues known to modify RGC axon growth [23], [31], [32]. These results indicated that the expression of *Pax6* in mouse RGCs prompt axon extension specifically in response to SFRP1, whereas it does not influence the response to Shh and Netrin1.

The Fz2 receptor appears to mediate the axonal response to SFRP1 in RGCs [14]. Thus, the selective induction of the expression of this receptor appeared as possible feasible mechanism of the action of Pax6. However, equal expression of Fz2 protein was detected in the axons of RGCs electroporated with either control or shRNAs targeting Pax6. Similarly quantitative PCR analysis did not reveal significant differences in the mRNA levels of *Fz2* from control and Pax6 overexpressing neurospheres or primary neurons (Fig. S4). Similarly, there were no significant changes in the expression of other members of the Frizzled receptor family expressed in the retina, [33], including Fz3, Fz5, Fz6 and Fz7 (Fig. S4). We therefore consider the possibility that Pax6 could regulate the expression of members of the family of Unc receptors, Unc5a, Unc5b, Unc5c and Unc5d, that contain SFRP1 binding domains [34] or that of other receptors implicated in axonal guidance like Neuropilin1 or PlexinD1 [35]. However, Pax6 overexpression in primary cortical cells did not induce significant changes in the expression of any of these genes (Fig. S4), suggesting that other factors might be involved.Hence, the regulation of the expression any of these receptors does not underlie the specific response to SFRP1 triggered by Pax6. Altogether, our results demonstrate that *Pax6* is necessary and sufficient to confer postmitotic differentiating neurons with the competence to respond to SFRP1, revealing a novel non neurogenic function of this pleiotropic transcription factor.

## Discussion

*Pax6* expression is crucial for dorsal-ventral and anterior-posterior patterning of the neural tube and controls neural precursor proliferation and fate decisions [13], [36], [37]. We herein demonstrated that expression of *Pax6* in postmitotic neurons is necessary and sufficient to provide axons with the competence to respond to SFRP1, a secreted molecule that can control growth cone movements [14]. This conclusion is based on the observation that abrogation or activation of Pax6 expression in neuronsthat, respectively, normally do or do not express Pax6, correlates with the response of their axons to SFRP1. This novel activity adds up to the very limited examples of Pax6 functions in postmitotic neurons, namely the control of dopaminergic neuron survival via the regulation of Crystallin αA [10]. In progenitors, other recent studies have also pointed out non-neurogenic roles of Pax6, such as the downregulation of the neurotrophin receptor p75^NTR^ [38]. Together, these studies consistently highlight alternative functions of this pleiotropic factor.

Although much progress has been made in defining the mechanisms directing the formation of brain circuitry, there is still much to learn about the precise coordination between axonal connectivity and the acquisition of neuronal identities, which, through the selective expression of certain specific transcription factors, specifies the interpretation of these signals. Indeed, the reported number of such transcription factors is still limited and needs to be expanded to obtain a truly comprehensive landscape of the determinants of neuronal connectivity. Moreover, the mechanisms that accurately synchronize acquisition of neuronal subtype identity with guidance molecule expression deserve further elucidation. Examples of this coordination are offered by the LIM homeodomain protein control of EphA receptor and ephrin-A ligand expression during motor neuron innervation of limb muscle [39]. Similarly, Lhx2/9 in the dorsal spinal cord directly regulates the expression of Rig-1 [40], a divergent member of the Robo family, required for midline crossing of commissural axons [41]. In the retina, expression of *Zic2* in the ventro-temporal retina determines ipsilateral identity of RGCs [42] and controls the behavior of their axons likely by regulating the expression of EphB1 [43], a specific guidance receptor implicated in ephrinB2-mediated repulsion of uncrossed axons at the optic chiasm midline [44]. However, genetic control of receptor expression is not always an evident explanation, and there are also multiple transcription factors shown to be critical in directing axonal trajectories by still unknown or alternative pathways. In the cerebral cortex for example, *Ctip2,* and *Satb2*, determine the final targets of corticospinal motor neurons and callosal projecting neurons, respectively [45], [46], [47], [48], [49] and recent studies report that these two cell populations differentially respond to Sema3A, a potent repellant of cortical axons [50]. Surprisingly, similar levels of Sema3A receptors are found in cells expressing Satb2 or Ctip2, and the selective response is proposed to be dependent on the control of the receptor's endocytic and signaling pathway [51].

In this scenario, our data provide an additional example of a transcription factor that selectively controls the response to axon guidance cues. Although we clearly demonstrated a relationship between Pax6 expression and response to SFRP1 in different neurons, we did not found an obvious mechanism to explain this relationship. In chick and *Xenopus*, SFRP1 binds to Fz2 to mediate axonal response [14]. Our experiments indicate the unlikely involvement of the Fz2 receptor and suggest other unknown mechanisms that we have been so far unable to decipher. There are several possible scenarios. Other yet undefined SFRP1 receptors or co-receptors might be involved. Pax6 might also control the expression of other molecules involved in the pathway downstream of SFRP1 response. Alternative Pax6 may regulate the expression of ECM molecules known to be needed to control SFRP1 response, such as laminin and fibronectin [14]. Previous microarray analysis of Pax6 mutants and cells over-expressing Pax6 makes way to these possibilities and have shown regulation of guidance molecules receptors such as the Netrin receptor Unc5h, PlexinA2 and EphA5, signal transduction molecules of the Wnt pathway such as Gpc3, or cell adhesion molecules such as tenascin C or cadherin 11 [52], [53]. Notably, the screening of a phage display peptide library revealed that SFRP1 binds with high affinity to the peptide motif L/V-VDGRW-L/V, which is present in Unc5H3 [34], although, whether the two proteins actually interact is still unknown.

Understanding the role of Pax6 and SFRP1 in the selective connectivity of neuronal subclasses may contribute to untangle a piece of the puzzle of brain circuits. In particular, in the retina, developing RGCs express different levels of Pax6 protein [11] and their axons travel in the vicinity of several anatomical domains of expression of SFRP1 that may serve as cue to establish their connectivity [15], [18], [54], [55]. In regard to other *Pax6* positive postmitotic populations in the brain, as the ones referred above, *Sfrp1* expression patterns would be consistent with a possible role of the Pax6-SFRP1 pair in circuit formation [16], [19]. However, although *Sfrp1* knock out mice have been generated and are viable, defects in axonal connectivity have not yet been investigated [16] and will require attention in the future, especially in relation to *Pax6,* as our results indicate.

Besides axon guidance, an additional interesting implication of our study is raised by the blocking experiments using anti-SFRP1 antibodies. Our data evidenced the secretion of endogenous SFRP1 by NSCs and differentiating primary cortical cells, which is consistent with the reported *Sfrp1* expression patterns in the developing and adult brain [19], [56]. NCS express Pax6 both *in vivo* and *in vitro*, which suggests possible roles for the coordinated functions of both molecules in the physiology of NSCs and neural niches.

## Methods

### Animals

All animal procedures were approved by the National Center for Biotechnology Animal Care and Use Committee, in compliance with National and European Legislation (ID 080016). C57BL6 mice were obtained from Harlan Laboratories, Inc. (Indianapolis, US). Morning of the day of the appearance of the vaginal plug was defined as embryonic day (E) 0.5.

### Antibodies, immunohistochemistry, histology

Mouse embryos were fixed with 4% paraformaldehyde (PFA) in 0.1 M phosphate-buffered saline (PBS; pH 7.4) and cryoprotected in 30% sucrose in PBS. 10–20 µm horizontal sections were produced and mounted on Superfrost plus microscope slides (Fisher Scientific, Pittsburgh, PA). Inmunostaining of tissue sections and explants, or cell cultures was performed as described [20] using the following antibodies: rabbit anti-GFP (Molecular probes, Eugene, OR, 1∶500), mouse anti-β-tubulin III (Sigma-Aldrich, ST Louis, MO, 1∶1000), rat anti-Bromodeoxiuridine (BrdU) (BD Biosciences, San Jose, CA, 1∶250), mouse anti-Islet-1/2 (39.4D5 1∶10) developed by T. Jessell and the monoclonal antibody anti-Nestin (Rat 401, 1∶100) developed by S. Hockfield, were obtained from the Development Studies Hybridoma Bank developed under the auspices of the NICHD and maintained by The University of Iowa, Department of Biological Sciences, Iowa City, IA 52242, rabbit anti-SFRP1 (Abcam, Cambridge UK, 1∶500), goat anti-Brn3 (C-13) (Santa Cruz Biotechnology, Inc., Santa Cruz, CA, 1∶250), rabbit anti-Caspase3 (Cell Signaling Tech Inc, Danuers, MA, 1∶500), rabbit anti-Pax6 (Covance, Princeton, NJ, 1∶500), mouse anti-MAP1b (Covance, Princeton, NJ, 1∶500), rabbit anti-Fz2 (Abcam, Cambridge, UK, 1∶200), mouse anti-GFAP (Sigma-Aldrich, ST Louis, MO, 1∶400), and Alexa Fluor 488-, 594- or 647-conjugated fluorescent secondary antibodies (Molecular probes, Eugene, OR, 1∶500).

### Epifluorescece and confocal microscopy

Confocal microscopy was performed with a TCS-SP5 (Leica Microsystems GmbH, Welzlar, Germany) Laser Scanning System. Tissue sections of 50 and 20 µm were analyzed by taking 0.2 µm serial optical sections with the LASAF v2.2.1 software (Leica Microsystems GmbH, Welzlar, Germany). Fluorescence microscopy was performed with a Leica DMRXA and images were captured with Leica CW4000 FISH Version V1.2 software.

### DNA constructs and recombinant proteins

The hairpin sequences targeting *Pax6* were: TRCN0000075377:ICCGGCCTGAAGCAAGAATACAGGTACTCGAGTACCTGTATTCTTGCTTCAGGTTTTTG;ITRCN0000075376:ICCGGCATGGCAAACAACCTGCCTATCTCGAGATAGGCAGGTTGTTTGCCATGTTTTTG;ITRCN0000075375:ICCGGGACGGCATGTATGATAAACTACTCGAGTAGTTTATCATACATGCCGTCTTTTTG;ITRCN0000075374:ICCGGCCACTTCAACAGGACTCATTTCTCGAGAAATGAGTCCTGTTGAAGTGGTTTTTG.

*Pax6* and green fluorescence protein (GFP) cDNAs were under the cytomegalovirus enhancer, chicken *β-actin* promoter, and rabbit *β-globin* poly (A) signal (CAG) (cytomegalovirus [CMV] and beta-actin) promoter. For knock-down experiments, a mixture of the selected shRNA lentiviral constructs (1 µg/µl) and pCAG-GFP (1 µg/µl) was used. For testing off-target effects, we co-electroporated a construct driving expression of a silent resistant form of *Pax6* (1 µg/µl; CAG-mut*Pax6*; see Text S1) and shRNA targeting *Pax6* (1 µg/µl) plasmids. *Pax6* lentiviral shRNA constructs were obtained from Sigma-Aldrich Inc. (St. Louis, MO). Purified recombinant SFRP1 protein was generated as previously described [21] and recombinant purified N-Shh (2.5 µg/ml) was a kind gift from Dr. Sebastian Pons [22].

### *In utero* and *ex vivo* electroporation

*In utero* and *ex vivo* electroporations were performed as described previously [23]. E13.5 pregnant C57BL6 female mice were anesthetized by continuous inhalation of Isoflurane (Baxter. Deerfield, US). The abdomen was opened and the uterine horns exposed. The DNA solution containing 0.03% fast green in PBS was injected into one eye of each embryo using a pulled glass micropipette. The head of each embryo was placed between tweezer type electrodes (CUY 650 P5 Nepa GENE, Chiba, Japan) and five square electric pulses (38V, 50 ms) were passed at 1 sec intervals using an Electro Square Porator ECM 830 (BTX, Harvard Apparatus, Holliston, MA, USA). The wall and skin of the abdominal cavity were sutured and the embryos were allowed to develop normally until E16-P0. For the *ex vivo* electroporation the head of the E13.5 embryos were transfer to a dissecting dish where they were electroporated with the above conditions. Then, electroporated retinas were dissected and incubated in DMEM-F12 (Gibco, Invitrogen, Carlsbad, CA) supplemented with N2 (Gibco, Invitrogen, Carlsbad, CA) medium at 37°C, 5% CO_2_ for 24 hours.

### Retinal explants and analysis of axonal growth

Dorsal retinal microexplants were plated on glass coverslips coated with poly-D-Lys (50 µg/ml; Sigma-Aldrich, Co. St. Louis, MO) and laminin (10 µg/ml; Invitrogen, Carlsbad, CA) and grown for 24 hours in DMEM-F12 medium supplemented with N2 in the presence or absence of SFRP1 (2 ng/µl) or N-Shh (1 ng/µl). After 24 hours, explants were fixed in 4% PFA at RT for 30 minutes and immunostained with anti-β-tubulin III and anti-GFP antibodies. Axonal length was determined in confocal images employing LaserPix v.4 image software (Bio-Rad). In non-electroporated explants, axonal length was estimated by counting the number of β-tubulin III positive axons crossing 600 and 900 µm radius circumferences of a minimum of 100 axons in each of three independent experiments (n = 3). For electroporated explants, we measured the length of each individual GFP positive axon from the edge of the explant up to the distal tip of the growth cone. Knock-down of *Pax6* in RGCs did not interfere with axonal initiation because the number of total axons per explants was not significantly different in control and *Pax6* shRNA electroporated retinas (not shown). It was also not affected by treatment with SFRP1, Netrin1 or Shh. For SFRP1 experiments at least 50 axons per condition in each independent experiments (n = 6) or 60 neurons per condition (n = 4) in Shh and Netrin1 experiments. We chose to specifically study SFRP1 response in dorsal explants because this population expresses high levels of Pax6 and is homogeneously composed of contra-laterally projecting axons facilitating the analysis of SFRP1 response.

### Neurosphere culture and nucleofection

Dorsal telencephalons of E13.5 WT embryos were dissected and cells were dissociated employing trypsin and a flamed-tip, glass Pasteur pipette. Dissociated cells were cultured in DMEM-F12 supplemented with N2 (Gibco, Invitrogen, Carlsbad, CA), heparin, recombinant human basic fibroblast growth factor (FGF-2) (10 ng/ml; R&D Systems, Minneapolis, MN), and epidermal growth factor (EGF; 10 ng/ml; Gibco, Invitrogen, Carlsbad, CA). Neurosphere cultures were passage every 3 days by mechanical dissociation. Dissociated cells were transfected using the nucleofector (Amaxa Biosystems, Gaithersburg, MD) with 5 µg of a 1∶1 mixture of CAG-GFP and CAG-Pax6 or CAG- empty vector. Nucleofection efficiency and GFP positive cells were analyzed by flow cytometer (Coulter Epix XL) and data were analyzed with the Win MIDI software. Nucleofected neurospheres were seeded on glass coverslips coated with poly-D-Lys and cultured in DMEM-F12 supplemented with N2, heparin and 1% fetal calf serum (FCS). Cells were fixed in 4% PFA at RT for 30 minutes, then immunostained with anti-β-tubulin III and anti-GFP antibodies. Axonal length was determined with the LaserPix v.4 image software (Bio-Rad, Richmond, CA) over images taken with a fluorescence microscopy Leica DMRXA. For analysis of the effects of *Pax6*, cells were fixed after 6 or 9 days in culture. To compare neurons that exit at the same time from the cell cycle, postmitotic neurons were labeled with a 12 h pulse of Bromodeoxiuridine (BrdU, Sigma-Aldrich, ST Louis, MO) (0.5 ng/ml) in the media and subsequently extensively washed. For blocking the endogenous SFRP1 activity a mix of anti-SFRP1 antibodies (4 µg/ml dialyzed in PBS): rabbit anti-SFRP-1 and goat anti-SFRP-1 (H-90 and C-19; Santa Cruz Biotechnology, Inc., Santa Cruz, CA) or a control IgG (4 µg/ml, dialyzed in PBS; rabbit anti-p-αPAK Ser 199/204; Santa Cruz Biotechnology, Inc., Santa Cruz, CA) were added to the culture media after nucleofection. For stimulation experiments, SFRP1 was added to the media 24 h after the nucleofection and the early axonal growth measured 3 days after the addition of SFRP1.

### Primary neurons culture and transfection

Dissociated cells from the dorsal telencephalon of E13.5 embryos were seeded onto 24 well Poly-D-Lys coated plates in Neurobasal media supplemented with B27 complement (Gibco, Invitrogen, Carlsbad, CA) and BrdU (0.5 ng/ml) to test proliferation. 24 h after seeding, cells where co-transfected with CAG-*Pax6* or the empty CAG- vector and CAG-GFP using lipofectamine 2000 (Invitrogen, Carlsbad, CA) and following manufacture procedure. Three days after transfection, cells were fixed with PFA 4%, and immunostained with anti-β-tubulin III and anti-GFP antibodies to measure their axonal length as describe next. GFP positive neurons did not incorporate BrdU demonstrating an effect on posmitotic neurons independent of cell cycle exit. As mentioned, SFRP1 was added to the media 24 h after transfection and the axons measured 2 days after the addition of the protein.

### Quantification of axonal length

In neurons differentiated from NSC or primary neurons, we identified the axon by morphological criteria and the specific staining of MAP1b, selectively localized to the distal part of axonal projections [24], but not in neurites. As illustrated in Fig. 1d, staining with MAP1b allowed us to identify the axon in primary neurons, while GFP staining reveals transfected cells. In neurons derived from NSCs after 6 and 9 days of differentiation, a long projection neurite was always clearly observed (Fig. 1c) and quantified as an axon. Specific staining with MAP1b demonstrated that the axon was always the longest neurite projection (99% Map1b positive projections were the longest neurite in the cell (>100 cells counted). Accordingly, experiments in which we measure MAP1b or the morphological identified neurite gave equivalent results (compared results shown in Fig. 1e and Fig. 2). We measured the axonal length from the nucleus of the neurons to the distal end of the axon where the growth cone is localized. We analyzed a minimum of 50 axons per condition in four independent experiments (n = 4).

### Statistical analysis

Quantitative results are expressed as the mean ± SD. Axonal length distributions of each population were compared using a Chi-square test. Differences in gene expression was analyzed were compared with Student's two-sample t test. P values are indicated in figure legends.

## Supporting Information

Figure S1 **Efficiency of** ***Pax6*** **overexpression and inhibition.** **a**) Transfection of pCAG-*Pax6* in NSCs results in efficient overexpression of Pax6 proteins in NSC derived neurons. Pax6 proteins are not expressed normally in GFP positive CAG-control targeted neurons, but are ectopically expressed (yellow) in cells transfected with CAG-*Pax6* plasmid. Bar indicates 20 µm. **b**) Fluorescent-activating cell sorting (FACs) analysis demonstrates more than 20% transfection efficiency. **c**) Ectopic Pax6 expression in NSCs does not alter the total number of secondary and primary neurites per cell. The number of neurites, excluding the axon, was quantified 9 days after transfection. **d**) Transfection of shRNA lentiviral constructs efficiently suppresses the ectopic expression of Pax6 in CHO cells. CHO cells co-trasfected with CAG-Pax6; control shRNA and CAG-GFP show expression of Pax6 protein (red). Pax6 is down-modulated in cells transfected with CAG-*Pax6* and shRNAs targeting *Pax6*. Number of cells >100 (n = 3). Bar indicates 30 µm. Graph represents the proportion of GFP positive cells expressing Pax6 protein. Data are expressed as the mean ± SD. (***) p<0.001.
(TIF)

Figure S2 **Ectopic expression of** ***Pax6*** **stimulates axonal growth in cortical neurons.** NSCs were nucleofected with CAG-empty vector or CAG-*Pax6* and co-electroporated with CAG-*GFP*. Graph shows the percentage of nucleofected neurons with respect to their axonal length after 6 days of differentiation. Results are equivalent to those obtained at 9 days. Over-expression of *Pax6* increments the axonal length compare with control neurons. Data are expressed as the mean ± SD. (**) p<0.01.
(TIF)

Figure S3 **SFRP1 stimulated axonal response in** ***Pax6*** **over-expressing neurons but not in the control population.** Graph represents the population distribution of neurons with axons longer than 100 µm or more. Data are expressed as the mean ± SD. (**) p<0.01; (***) p<0.001.
(TIF)

Figure S4 **Knock-down of** ***Pax6*** **did not affect the expression of Fz2 receptor.** **a**) Micrographs show confocal images of GFP positives axons in retina explants that were electroporated with shRNA control or shRNA targeting *Pax6,* and with CAG-GFP. Both populations present low levels of Fz2 expression and a positive dotted pattern of Fz2 staining. GFP negative mature axons presented a stronger signal. Bar indicates 3 µm. **b**) Q-PCR detection of the relative expression of the mRNA of Fz1, Fz3, Fz5, Fz6, Unc5a, Unc5b, Unc5c, Unc5d, Neuropilin1 and PlexinD1 from primary cultured neurons. Expression levels are relative to GAPDH transcript and normalized to one control sample (see Text S2). There are no differences in the relative mRNA expression of these receptors in control and Pax6 transfected cells.
(TIF)

Text S1 **cDNA sequence for the silent mutant form of** ***Pax6*** **resistant.**
(DOCX)

Text S2 **Methods for real-time quantitative RT-PCR (Q-PCR).**
(DOCX)
