# Supplementary figures and images for: Pαx6 Expression in Postmitotic Neurons Mediates the Growth of Axons in Response to SFRP1
Source: PLoS One. 2012 Feb 16;7(2):e31590. doi: 10.1371/journal.pone.0031590 (PMC3281087; doi:10.1371/journal.pone.0031590)

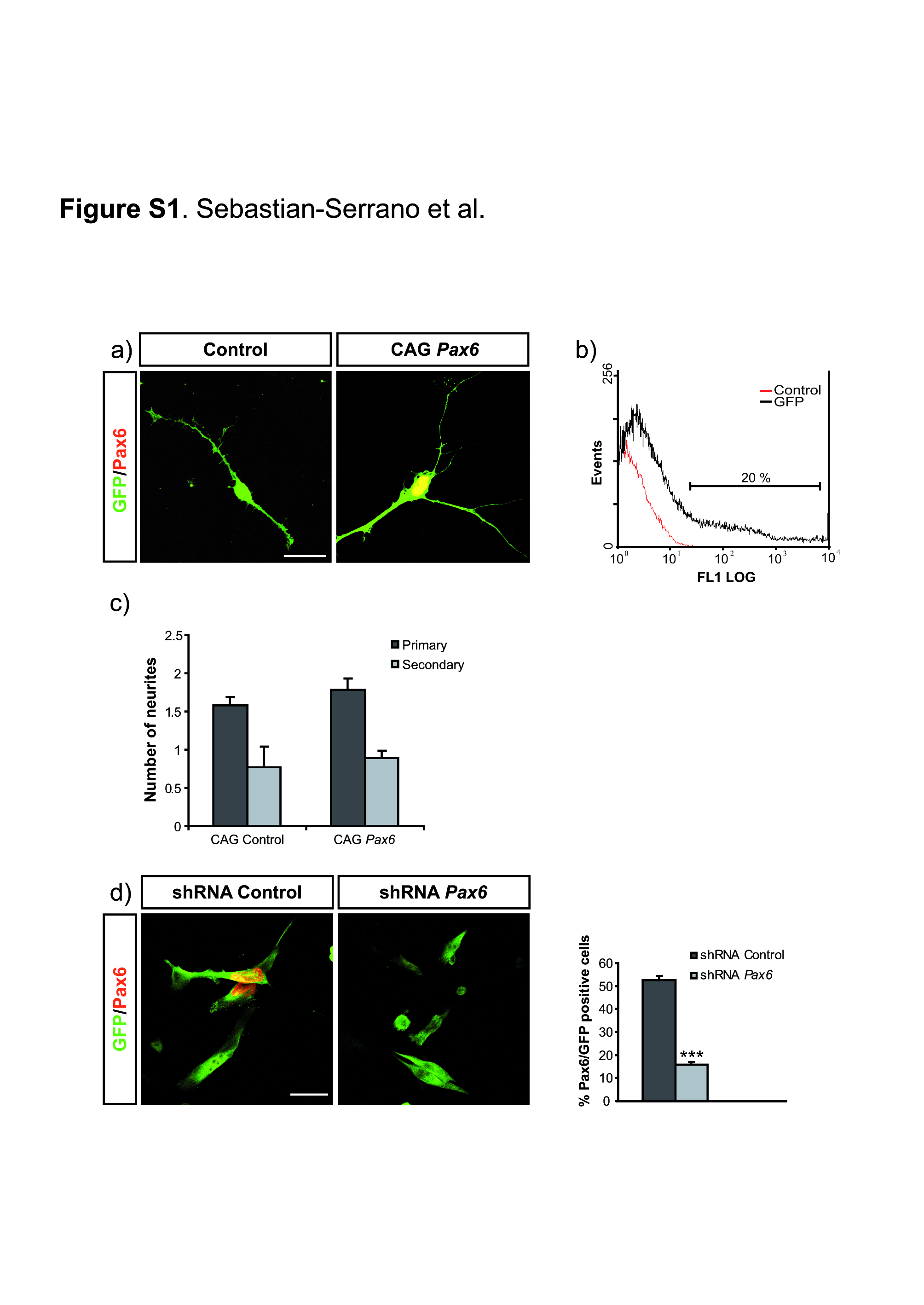

Supplement: Figure S1 — Efficiency of Pax6 overexpression and inhibition. a) Transfection of pCAG-Pax6 in NSCs results in efficient overexpression of Pax6 proteins in NSC derived neurons. Pax6 proteins are not expressed normally in GFP positive CAG-control targeted neurons, but are ectopically expressed (yellow) in cells transfected with CAG-Pax6 plasmid. Bar indicates 20 µm. b) Fluorescent-activating cell sorting (FACs) analysis demonstrates more than 20% transfection efficiency. c) Ectopic Pax6 expression in NSCs does not alter the total number of secondary and primary neurites per cell. The number of neurites, excluding the axon, was quantified 9 days after transfection. d) Transfection of shRNA lentiviral constructs efficiently suppresses the ectopic expression of Pax6 in CHO cells. CHO cells co-trasfected with CAG-Pax6; control shRNA and CAG-GFP show expression of Pax6 protein (red). Pax6 is down-modulated in cells transfected with CAG-Pax6 and shRNAs targeting Pax6. Number of cells >100 (n = 3). Bar indicates 30 µm. Graph represents the proportion of GFP positive cells expressing Pax6 protein. Data are expressed as the mean ± SD. (***) p<0.001. (TIF) [file pone.0031590.s001.tif]

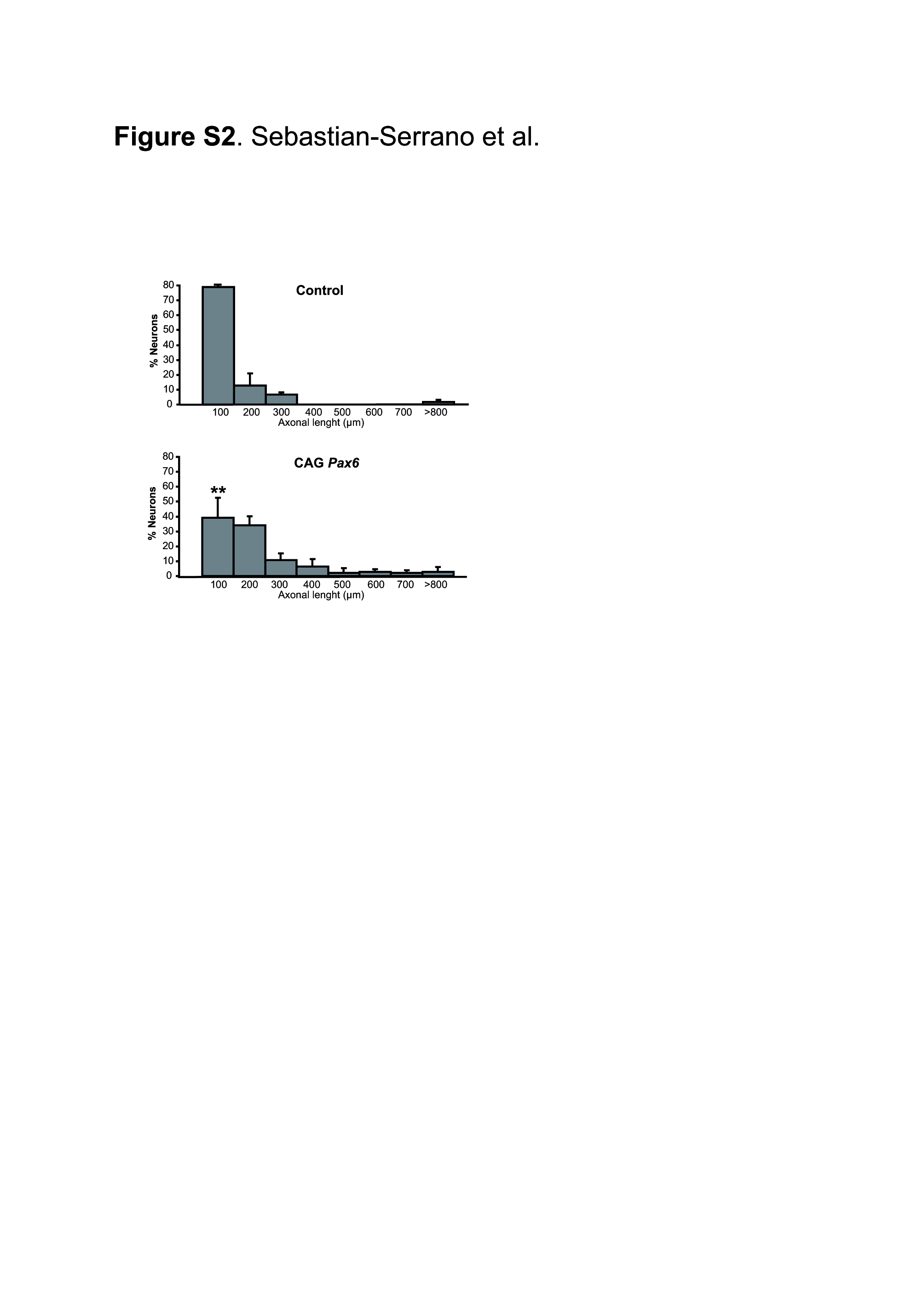

Supplement: Figure S2 — Ectopic expression of Pax6 stimulates axonal growth in cortical neurons. NSCs were nucleofected with CAG-empty vector or CAG-Pax6 and co-electroporated with CAG-GFP. Graph shows the percentage of nucleofected neurons with respect to their axonal length after 6 days of differentiation. Results are equivalent to those obtained at 9 days. Over-expression of Pax6 increments the axonal length compare with control neurons. Data are expressed as the mean ± SD. (**) p<0.01. (TIF) [file pone.0031590.s002.tif]

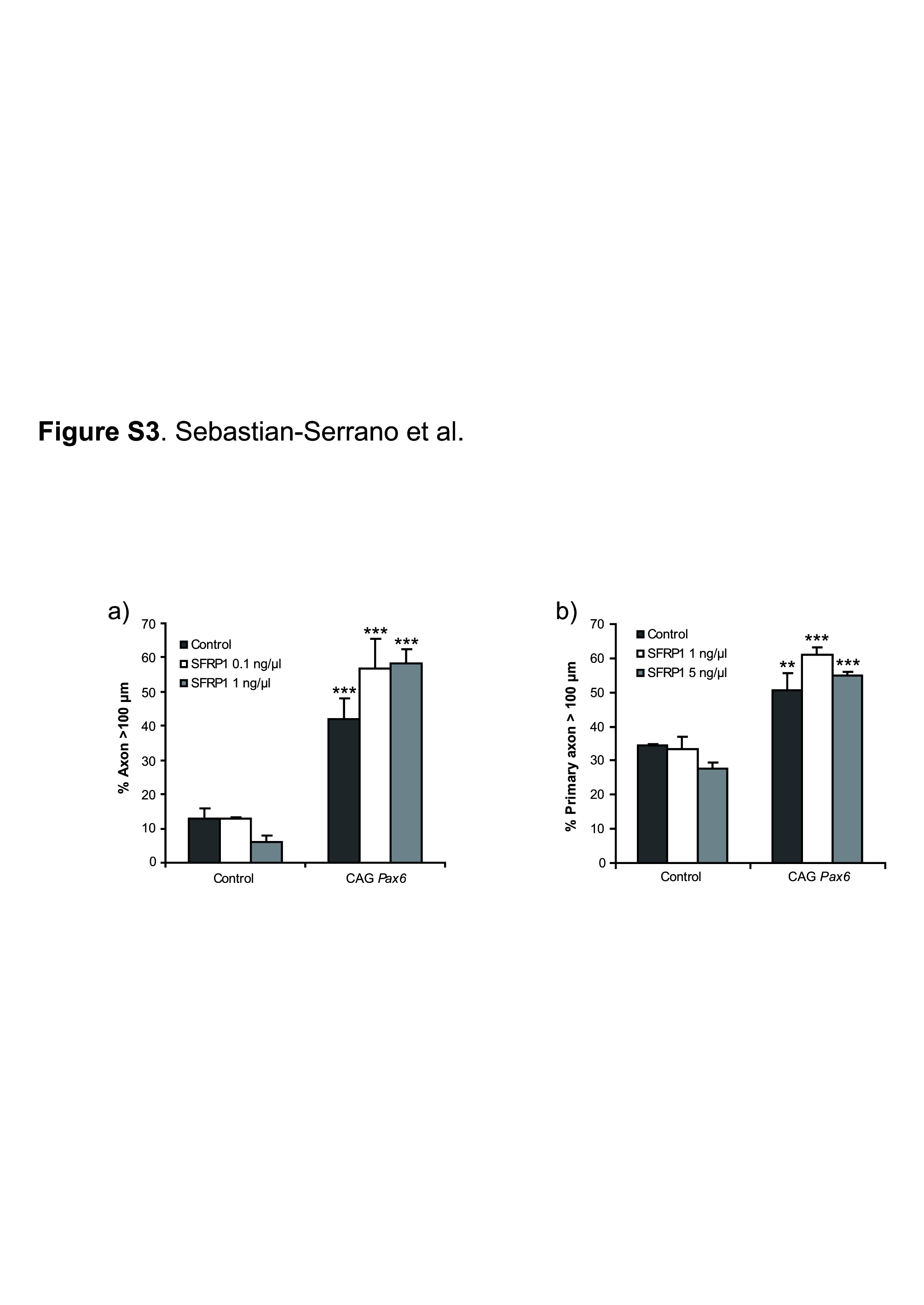

Supplement: Figure S3 — SFRP1 stimulated axonal response in Pax6 over-expressing neurons but not in the control population. Graph represents the population distribution of neurons with axons longer than 100 µm or more. Data are expressed as the mean ± SD. (**) p<0.01; (***) p<0.001. (TIF) [file pone.0031590.s003.tif]

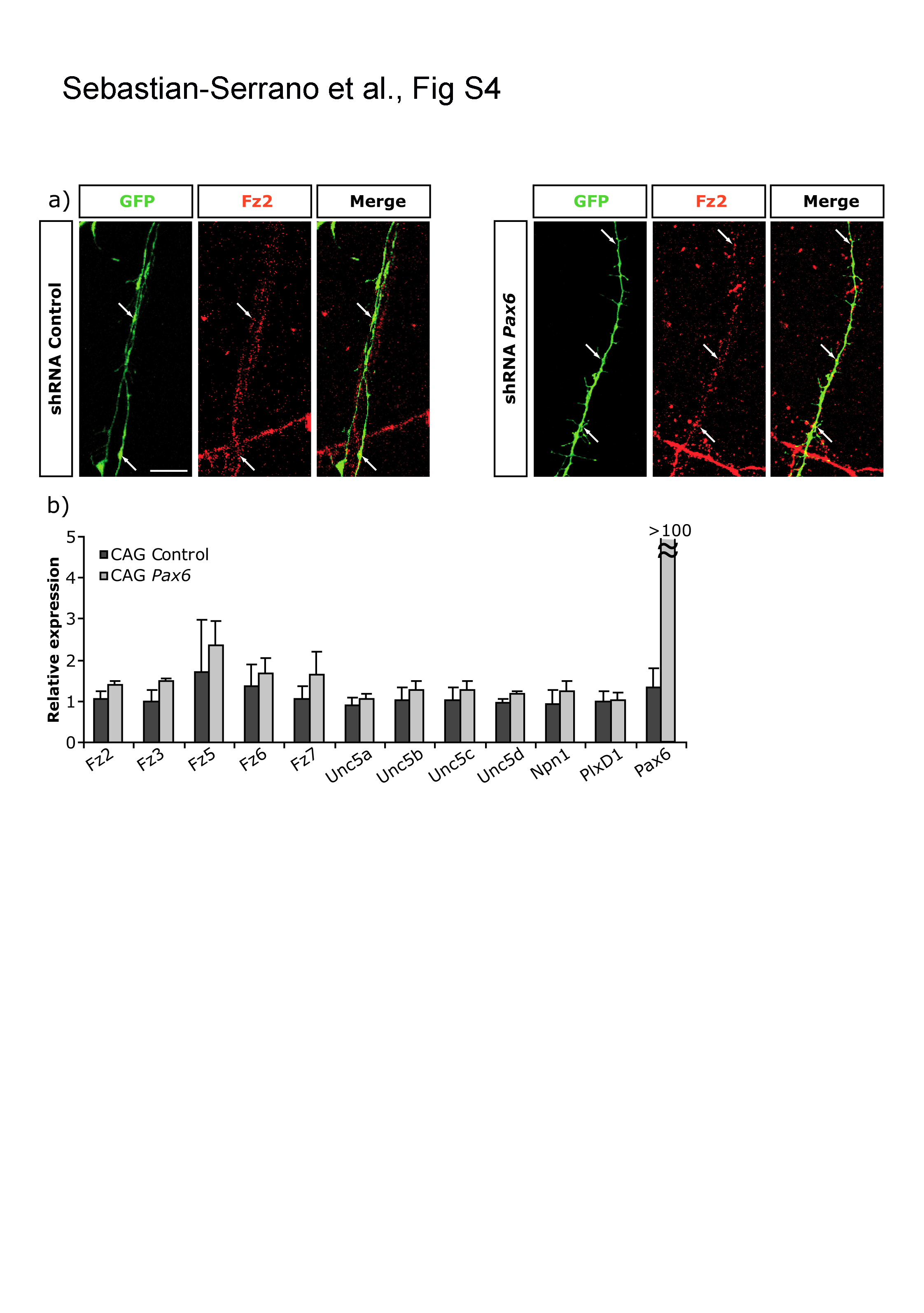

Supplement: Figure S4 — Knock-down of Pax6 did not affect the expression of Fz2 receptor. a) Micrographs show confocal images of GFP positives axons in retina explants that were electroporated with shRNA control or shRNA targeting Pax6, and with CAG-GFP. Both populations present low levels of Fz2 expression and a positive dotted pattern of Fz2 staining. GFP negative mature axons presented a stronger signal. Bar indicates 3 µm. b) Q-PCR detection of the relative expression of the mRNA of Fz1, Fz3, Fz5, Fz6, Unc5a, Unc5b, Unc5c, Unc5d, Neuropilin1 and PlexinD1 from primary cultured neurons. Expression levels are relative to GAPDH transcript and normalized to one control sample (see Text S2). There are no differences in the relative mRNA expression of these receptors in control and Pax6 transfected cells. (TIF) [file pone.0031590.s004.tif]
