# Supplementary material for: Pαx6 Expression in Postmitotic Neurons Mediates the Growth of Axons in Response to SFRP1
Source: PLoS One. 2012 Feb 16;7(2):e31590. doi: 10.1371/journal.pone.0031590 (PMC3281087; doi:10.1371/journal.pone.0031590)
Supplement: Text S1 — cDNA sequence for the silent mutant form of Pax6 resistant. (DOCX) [file pone.0031590.s005.docx]

**Text S1.**

The cDNA sequence for the silent mutant form of *Pax6* resistant form was **:** GCG CTC GAGCCACCACCATGCAGAACAGTCACAGCGGAGTGAATCAGCTTGGTGGTGTCTTTGTCAACGGGCGGCCACTGCCGGACTCCACCCGGCAGAAGATCGTAGAGCTAGCTCACAGCGGGGCCCGGCCGTGCGACATTTCCCGAATTCTGCAGACCCATGCAGATGCAAAAGTCCAGGTGCTGGACAATGAAAACGTATCCAACGGTTGTGTGAGTAAAATTCTGGGCAGGTATTACGAGACTGGCTCCATCAGACCCAGGGCAATCGGAGGGAGTAAGCCAAGAGTGGCGACTCCAGAAGTTGTAAGCAAAATAGCCCAGTATAAACGGGAGTGCCCTTCCATCTTTGCTTGGGAAATCCGAGACAGATTATTATCCGAGGGGGTCTGTACCAACGATAACATACCCAGTGTGTCATCAATAAACAGAGTTCTTCGCAACCTGGCTAGCGAAAAGCAACAGATGGGCGCAgatgggatgtacgATAAACTAAGGATGTTGAACGGGCAGACCGGAAGCTGGGGCACACGCCCTGGTTGGTATCCCGGGACTTCAGTACCAGGGCAACCCACGCAAGATGGCTGCCAGCAACAGGAAGGAGGGGGAGAGAACACCAACTCCATCAGTTCTAACGGAGAAGACTCGGATGAAGCTCAGATGCGACTTCAGCTGAAGCGGAAGCTGCAAAGAAATAGAACATCTTTTACCCAAGAGCAGATTGAGGCTCTGGAGAAAGAGTTTGAGAGGACCCATTATCCAGATGTGTTTGCCCGGGAAAGACTAGCAGCCAAAATAGATCTAcccgaggccaggattcaagtgTGGTTTTCTAATCGAAGGGCCAAATGGAGAAGAGAAGAGAAACTGAGGAACCAGAGAAGACAGGCCAGCAACACTCCTAGTCACATTCCTATCAGCAGCAGCTTCAGTACCAGTGTCTACCAGCCAATCCCACAGCCCACCACACCTGTCTCCTCCTTCACATCAGGTTCCATGTTGGGCCGAACAGACACCGCCCTCACCAACACGTACAGTGCTTTGCCACCCATGCCCAGCTTCACCatggcgaataatctccccatGCAACCCCCAGTCCCCAGTCAGACCTCCTCATACTCGTGCATGCTGCCCACCAGCCCGTCAGTGAATGGGCGGAGTTATGATACCTACACCCCTCCGCACATGCAAACACACATGAACAGTCAGCCCATGGGCACCTCGGGGAcaacatctaccggccttatatcACCTGGAGTGTCAGTTCCCGTCCAAGTTCCCGGGAGTGAACCTGACATGTCTCAGTACTGGCCTCGATTACAGTA AAA ATG CGG CCG CTC TTT ACT.
