# Supplementary material for: Pαx6 Expression in Postmitotic Neurons Mediates the Growth of Axons in Response to SFRP1
Source: PLoS One. 2012 Feb 16;7(2):e31590. doi: 10.1371/journal.pone.0031590 (PMC3281087; doi:10.1371/journal.pone.0031590)
Supplement: Text S2 — Methods for real-time quantitative RT-PCR (Q-PCR). (DOCX) [file pone.0031590.s006.docx]

**Text S2.**

**Real-time quantitative RT-PCR (Q-PCR).** 1 μg of total RNA from cells was reverse transcribed with random primers (Invitrogen) and the superscript reverse transcriptase (Life Technologies) to obtain cDNA. PCR reaction mixtures containing cDNA and DNA Master Sybr green I mix (Applied Biosystems) were incubated at 95ºC for 5 min followed by 40 PCR cycles (5s at 95ºC, 45s at 60ºC, 90s at 68ºC) in an Abi-prism 7000 detector (Applied Biosystems). Specific primers for *Fz2* were 5’-TCCTCACATGGTCGGTGTTG-3’, 5’- CGCTGCATGTCCACTAAATAGG-3’. The results were normalized as indicated by the parallel amplification of GADPH (5'-TGACGTGCCGCCTGGAGAAA-3', 5'-AGTGTAGCCCAAGATGCCCTTCAG -3').
